# Generative Adversarial Domain Adaptation for Nucleus Quantification in Images of Tissue Immunohistochemically Stained for Ki-67

**DOI:** 10.1200/CCI.19.00108

**Published:** 2020-07-30

**Authors:** Xuhong Zhang, Toby C. Cornish, Lin Yang, Tellen D. Bennett, Debashis Ghosh, Fuyong Xing

**Affiliations:** ^1^ Department of Biostatistics and Informatics, University of Colorado Anschutz Medical Campus, Aurora, CO; ^2^ Department of Pathology, University of Colorado Anschutz Medical Campus, Aurora, CO; ^3^ Department of Electrical and Computer Engineering, Department of Computer and Information Science, Department of Biomedical Engineering, University of Florida, Gainesville, FL; ^4^ Department of Pediatrics, University of Colorado Anschutz Medical Campus, Aurora, CO; ^5^ The Data Science to Patient Value Initiative, University of Colorado Anschutz Medical Campus, Aurora, CO

## Abstract

**PURPOSE:**

We focus on the problem of scarcity of annotated training data for nucleus recognition in Ki-67 immunohistochemistry (IHC)–stained pancreatic neuroendocrine tumor (NET) images. We hypothesize that deep learning–based domain adaptation is helpful for nucleus recognition when image annotations are unavailable in target data sets.

**METHODS:**

We considered 2 different institutional pancreatic NET data sets: one (ie, source) containing 38 cases with 114 annotated images and the other (ie, target) containing 72 cases with 20 annotated images. The gold standards were manually annotated by 1 pathologist. We developed a novel deep learning–based domain adaptation framework to count different types of nuclei (ie, immunopositive tumor, immunonegative tumor, nontumor nuclei). We compared the proposed method with several recent fully supervised deep learning models, such as fully convolutional network-8s (FCN-8s), U-Net, fully convolutional regression network (FCRN) A, FCRNB, and fully residual convolutional network (FRCN). We also evaluated the proposed method by learning with a mixture of converted source images and real target annotations.

**RESULTS:**

Our method achieved an *F*_1_ score of 81.3% and 62.3% for nucleus detection and classification in the target data set, respectively. Our method outperformed FCN-8s (53.6% and 43.6% for nucleus detection and classification, respectively), U-Net (61.1% and 47.6%), FCRNA (63.4% and 55.8%), and FCRNB (68.2% and 60.6%) in terms of *F*_1_ score and was competitive with FRCN (81.7% and 70.7%). In addition, learning with a mixture of converted source images and only a small set of real target labels could further boost the performance.

**CONCLUSION:**

This study demonstrates that deep learning–based domain adaptation is helpful for nucleus recognition in Ki-67 IHC stained images when target data annotations are not available. It would improve the applicability of deep learning models designed for downstream supervised learning tasks on different data sets.

## INTRODUCTION

Neuroendocrine tumors (NETs) are heterogeneous cancers that affect most organ systems. The incidence of NETs is increasing, with approximately 12,000 new diagnoses in the United States each year.^1^ The 5-year survival rate of patients with NETs is associated with tumor grades^2^ determined by the proliferation rate of the neoplastic cells, most commonly by measuring the Ki-67 labeling index (LI).^3-5^ Accurate grading of NETs is necessary to ensure proper treatment and patient management. Measurement of the Ki-67 LI from pathology images requires accurate cell/nucleus classification (ie, quantification of immunopositive and immunonegative tumor cells while excluding nontumor cells). This is an essential procedure in basic, translational, and clinical research and in routine clinical practice. However, the commonly used “eyeball” estimation method for Ki-67 counting often leads to poor reliability and reproducibility, and manual counting is inefficient and subjective.^6-8^ To address these issues, computerized methods, including machine learning–based algorithms, have been introduced to quantify different types of cells.^9^ In particular, deep learning has drawn considerable attention in digital pathology and microscopy image analysis.^10^

**CONTEXT**

**Key Objective**
To develop an adversarial learning-based domain adaptation method to count different types of nuclei for automated Ki-67 labeling index assessment.
**Knowledge Generated**
Without any target data annotations, adversarial learning-based domain adaptation is able to conduct automated nucleus recognition for Ki-67 scoring in Ki-67 immunohistochemistry–stained target images. In addition, learning a deep model with a mixture of source images and only little real target annotation can further improve model performance.
**Relevance**
The proposed method can address the issue of image appearance variation in staining by using generative adversarial learning such that it would significantly improve the re-use of state-of-the-art deep learning algorithms for Ki-67 scoring in clinical research and practice. In addition, it provides a pixel-to-pixel learning pipeline for automated, single-stage nucleus detection and classification and thus, could eliminate the need for pathologists to exclude areas of nonrelevant regions for Ki-67 image analysis.

Deep neural networks are emerging as a powerful tool for a wide variety of computer vision tasks,^11,12^ including biomedical image computing.^13,14^ Currently, convolutional neural networks (CNNs)^15,16^ are the dominant deep learning technology for various biomedical image analysis applications.^10,17,18^ CNNs have been applied to nucleus detection^19^ and im-age segmentation^20^ in Ki-67–stained pancreatic NET images; however, few studies have proposed deep learning–based Ki-67 counting. Although a CNN-based approach^21^ has been applied to differentiation between immunopositive and immunonegative tumor nuclei, it might not exclude nontumor nuclei for Ki-67 counting. A recent report^22^ has introduced a deep fully convolutional network (FCN) for single-stage nucleus recognition for Ki-67 counting in pancreatic NETs, and the network allows for simultaneous nucleus detection and classification by using pixel-to-pixel modeling. Another end-to-end CNN,^23^ which requires a prerequisite of individual cell segmentation, has been applied to cell classification in breast cancer Ki-67 images. Both methods provide excellent nucleus/cell classification and outperform other machine learning–based approaches, which shows the great potential of deep learning in Ki-67 LI assessment. However, they as well as other CNN-based methods often require a large number of annotated training images. Medical image annotation is often labor intensive, especially individual nucleus labeling as required for Ki-67 scoring. In real applications, there might be few labeled data in one specific data set but a sufficient number of labeled images in another (eg, other imaging sources). However, models trained on one data set might not be directly applicable to another because of data set shift, a situation where the joint distribution of inputs and outputs differs between the training and test stages. We hypothesize that deep learning–based domain adaptation, which can transfer learned knowledge from existing data sets to others, is helpful for nucleus recognition such that deep models can be reused for different data sets.

**FIG 1.**
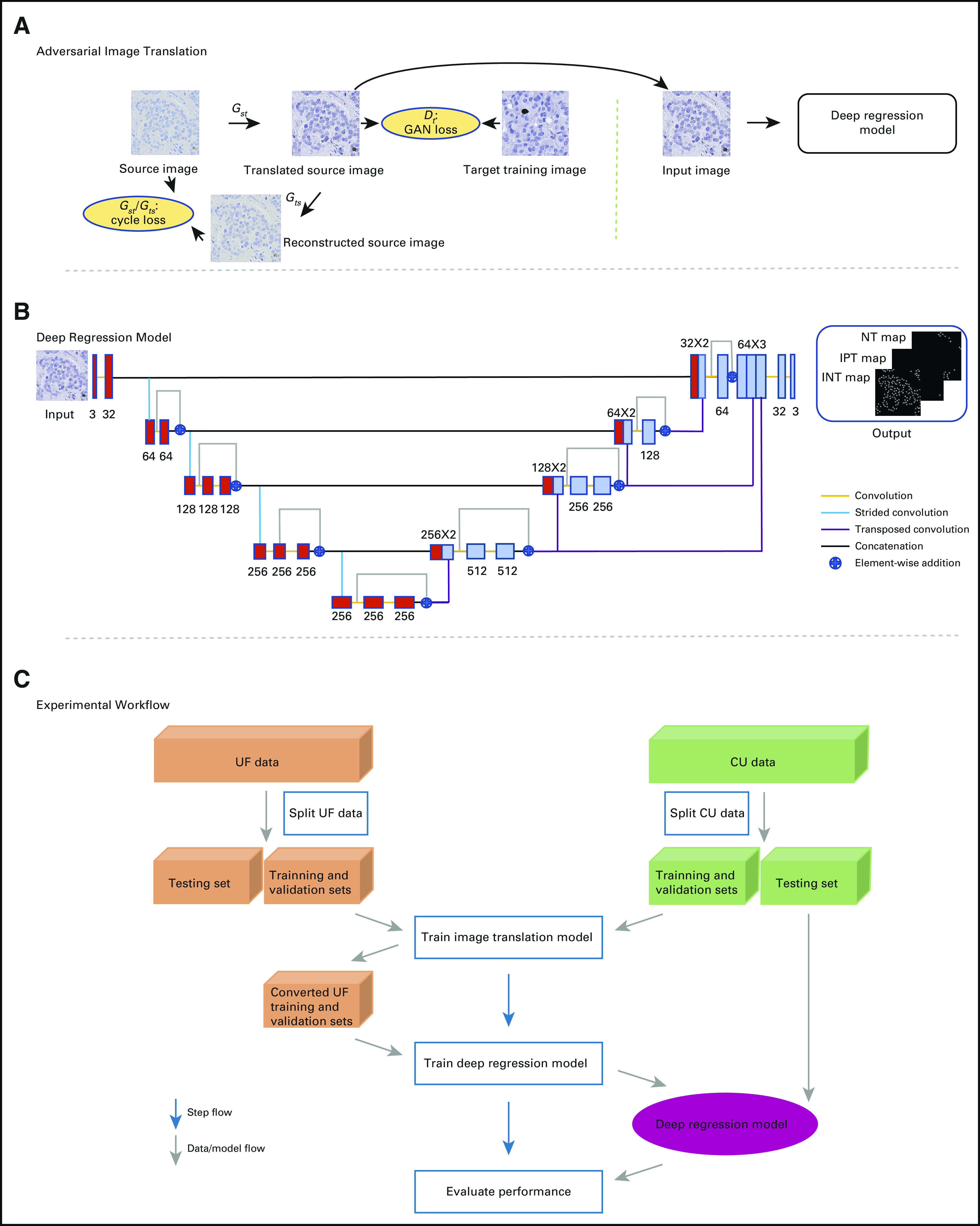
Overview of the proposed framework. (A) Adversarial image translation. *G_st_* and *D_t_* are the source-to-target generator and its associated discriminator, respectively. *G_ts_* is the target-to-source generator. The generative adversarial network (GAN) loss and the cycle loss are used to train the GANs. Here, source and target images are from the University of Florida (UF) and University of Colorado (CU), respectively. (B) Deep regression model. The red and light blue boxes denote feature maps at different levels. The number of feature maps in each layer is shown above or below the boxes. Different colors denote different operations. (C) Experimental workflow. INT, immunonegative tumor; IPT, immunopositive tumor; NT, nontumor.

In this study, we developed a novel deep learning–based domain adaptation framework (Fig 1) to quantify nuclei for Ki-67 LI assessment in pancreatic NETs. This framework can convert Ki-67 immunohistochemistry (IHC)-stained images from an existing, annotated data set (ie, source) to another style of images that look similar to those in an unannotated or limited annotated data set (ie, target) in terms of color and texture. Thus, it enables nucleus recognition in the target data set if no target data annotations are available. Specifically, this framework learns a cycle-consistent generative adversarial network (GAN)^24,25^ (see Appendix, Explanation of Terminology/Algorithms, for detailed descriptions of this term and others) for image conversion between source and target data sets and then trains a deep regression model with the converted source images and corresponding annotations to locate and classify different types of nuclei in the target data set. In this scenario, the framework is able to significantly reduce human effort for data annotation by eliminating the need for additional annotation of images in the target data set, thereby shortening the period of algorithm development.

## METHODS

### Data Sets

We collected pancreatic NET image data sets from 2 different academic medical centers: University of Florida (UF) and University of Colorado (CU). Additional details about cohort assembly are provided in the Data Collection section of the Appendix. Briefly, the UF data set contained 38 cases of IHC Ki-67–stained tissue microarray (TMA) images captured at 20× magnification, and each case had three 500 × 500 × 3 (ie, width × height × number of image channels in pixels) images cropped from TMA cores (114 total images). Each image had individual nucleus annotations available (ie, position and category [immunopositive tumor, immunonegative tumor, nontumor]). The CU data set contained 72 cases of IHC Ki-67–stained whole-slide imaging (WSI) data captured at 40× magnification. Each case had 1 WSI slide from which an approximately 1,192 × 1,192 × 3 (ie, width × height × number of image channels in pixels) image was cropped (72 total images). The cropped images were annotated by an expert pancreatic pathologist using a custom tool developed in MATLAB (MathWorks, Natick, MA). Each nucleus in an image was assigned to 1 of the 3 classes by placing a marker as near to the nuclear center as possible.

### Adversarial Image Translation

An overview of the proposed framework is shown in Fig 1A. To reduce the variability of image appearance between the data sets (ie, source, target), we applied generative adversarial learning^24^ to image translation in a pixel-level space such that converted/adapted source images looked like those in the target data set. Compared with domain adaptation in a feature space, pixel-level translation is more suitable for structured prediction tasks,^26,27^ such as nucleus localization and categorization. To better preserve image content during image-to-image translation, we introduced a cycle-consistent constraint^25,27a^ into the adversarial learning.

Formally, let (*X^s^*,*Y^s^*) represent the training images (*X^s^*) and associated annotations/labels (*Y^s^*) in the source data set, and *X^t^* denote the unannotated training images in the target data set. By using a cycle-consistent GAN (see Appendix for mathematical equation) that consists of 2 generator-discriminator pairs (*G_st_*, *D_t_*) and (*G_ts_*, *D_s_*), we aimed to translate source images *X^s^* into target-like ones *G_st_*(*X^s^*) such that the discriminator *D_t_* is unable to differentiate *G_st_*(*X^s^*) and *X^t^*. In our implementation, the generators and discriminators were selected as a 9–residual-learning-block FCN^28^ and a 70 × 70 PatchGAN,^29^ respectively.

### Deep Regression Model

With adversarial image translation, the adapted source images appeared as if drawn from the target data set, but the content was preserved. A model trained with the adapted source images and associated annotations can therefore be applied to nucleus recognition on real target images. We then trained a U-Net–like regression model (Fig 1B), which was built on a deep structured prediction network.^22^ Instead of using 2 branches to identify nuclei and requiring additional region of interest (ROI) annotations,^22^ our model adopted only 1 branch for a single task requir-ing no ROI labeling. In addition, we did not penalize the correlation between different feature maps in higher layers but directly used 2 convolutional layers for nucleus identification (Fig 1B). This strategy can reduce memory usage and accelerate model training.

Specifically, our deep regression model (see Appendix for the mathematical equation) is a variant of an encoder-decoder network architecture, U-Net,^30^ which has multiple long-range skip connections between the encoder and decoder. In our design, the encoder and decoder consist of 4 stacked residual learning blocks.^31^ In addition, we fused the information from different layers such that the model can handle scale variation of nuclei.^22^ The fused information was finally fed into 2 consecutive convolutional layers for output prediction. During training, we used both converted and original source images for better learning.^32^ During testing, we applied the learned regressor *R* to output map prediction on new target images. For each channel of output map  ŷ, we suppressed pixel values < *η* ⋅ *max* (ŷ), where *η* ∈ [0,1], and sought local maxima as the detected nucleus centers, whose labels were determined by finding the largest value across the 3 channels of ŷ.

### Experimental Setup and Evaluation Metrics

We randomly split each data set into training (50%) and test (50%) sets at the case level, and selected 20% of training data as the validation set (Fig 1C). We chose the UF data set as the source because all 114 images were labeled. The CU data set was the target. We conducted twofold cross-validation. More training details are explained in the Appendix.

We evaluated the proposed method for nucleus detection and classification. For nucleus detection, we merged the 3 channels of the output prediction map by taking the largest values for each pixel across the channels and found local maxima as nucleus centers.^22^ For each annotation point, we defined its gold-standard area as a circular region with radius *r* = 16 pixels centered at that point.^22,33^ Within gold-standard areas, the detected nucleus centers were associated with corresponding annotations using the Hungarian algorithm.^34^ Each annotation had at most 1 detection point and vice versa. The detection points that matched gold-standard annotations were considered true positives (TPs), and all others were false positives (FPs). The annotations without any associated detections were viewed as false negatives (FNs). We quantified the nucleus detection performance with precision (P), recall (R), and *F*_1_ score as follows: P = TP/(TP + FP), R = TP/(TP + FN), and *F*_1_ = 2PR/(P + R). We also reported the area under the precision-recall curve (AUC), which was generated by varying *η* from 0 to 1. For nucleus classification evaluation, we calculated the weighted average precision, recall, *F*_1_ score, and AUC across the 3 categories of nuclei,^22,35^ and the weight was the percentage of each nucleus subtype in the test set. In the experiments, we also evaluated the effects of the radius *r*, which is used to define the gold-standard area, on nucleus recognition.

### Data Availability Statement

This study was approved by the CU Anschutz Medical Campus institutional review board (#17-2167). Requests for the data sets used in this study should be addressed to the corresponding author. The source codes can be accessed through GitHub.^36^

## RESULTS

**FIG A1.**
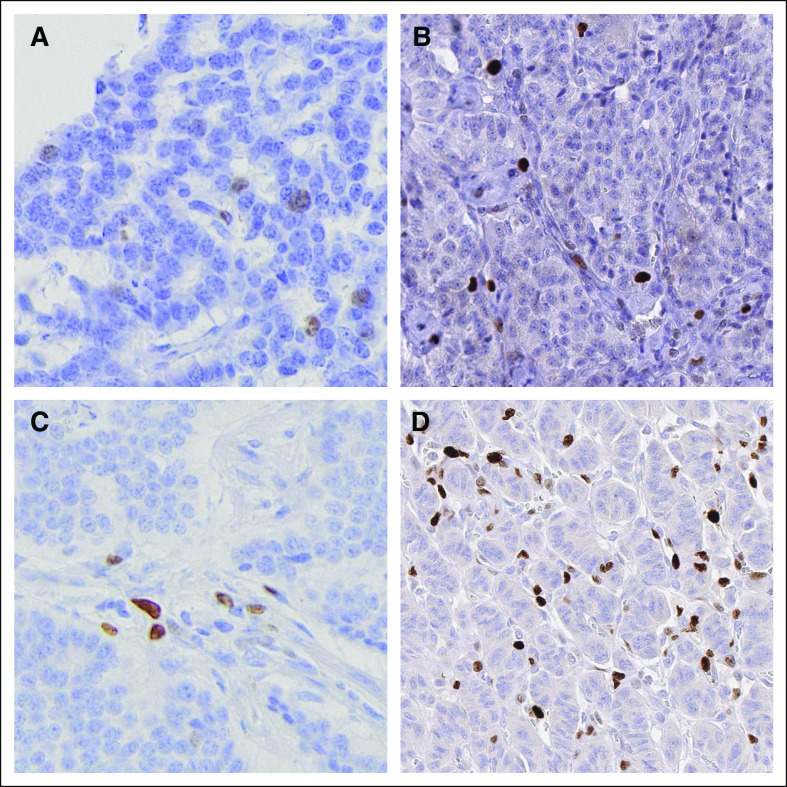
Examples of color variability in images from (A and C) the University of Florida and (B and D) the University of Colorado.

The UF data set consisted of 114 images from 38 patients with 22,198 nuclei in total (1,217 immunopositive tumors, 15,529 immunonegative tumors, and 5,452 nontumor nuclei). The CU data set contained 72 images from 72 patients. Twenty CU images were annotated, with 11,780 nuclei annotated (1,519 immunopositive tumor, 7,989 immunonegative tumor, and 2,272 nontumor nuclei). Although both data sets were Ki-67 IHC stained, they exhibited significant variability of image appearance (Appendix Fig A1). Table 1 lists the characteristics of patients in the CU data set.

**TABLE 1.**
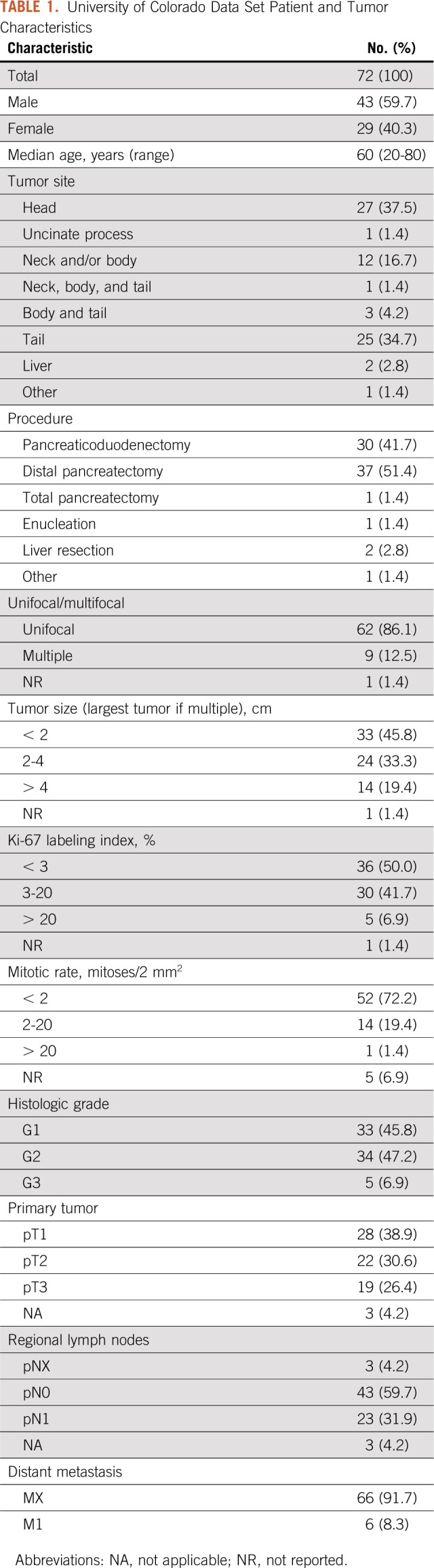
University of Colorado Data Set Patient and Tumor Characteristics

**FIG 2.**
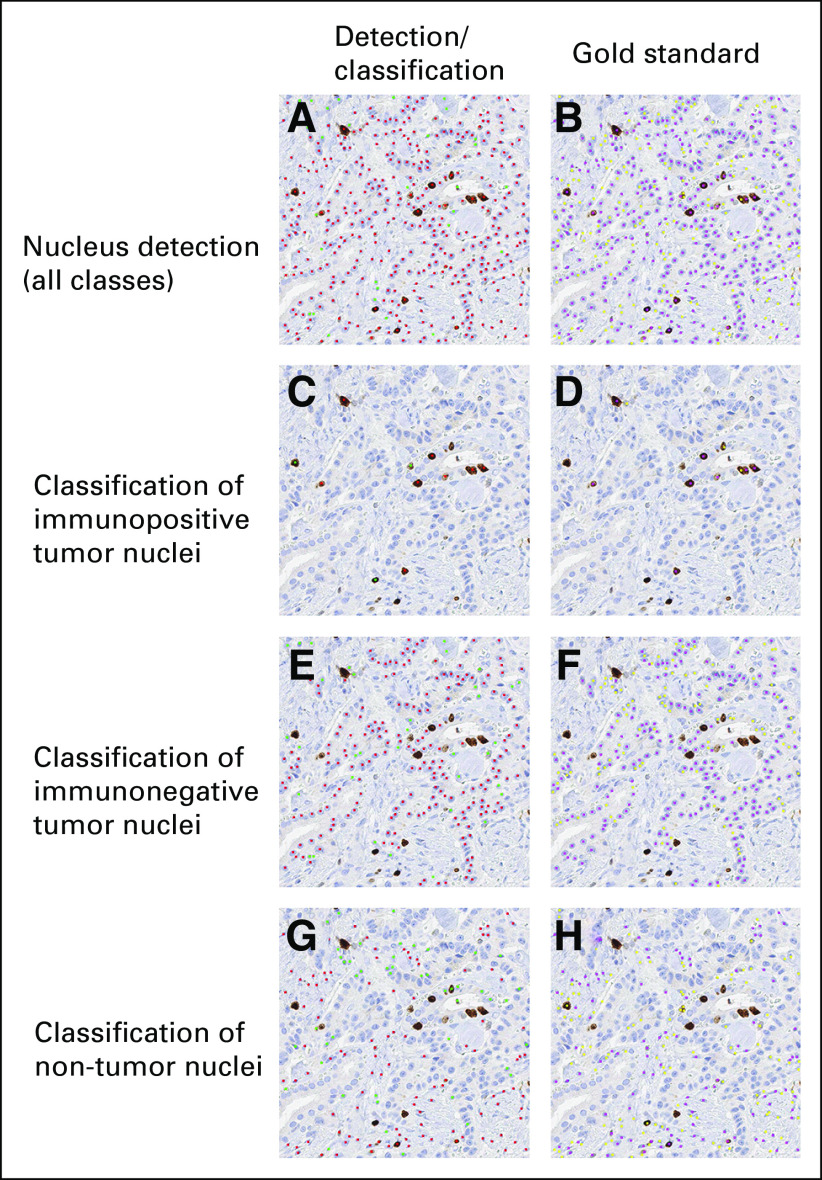
Qualitative results of nucleus detection and classification on the University of Colorado (CU) data. The left and right columns represent model predictions and gold-standard annotations, respectively. (A and B) Nucleus detection results, with 374 true positives (TPs), 30 false positives (FPs), 155 false negatives (FNs), and 354,657 true negatives (TNs). (C and D) Nucleus classification results for immunopositive tumor, (E and F) immunonegative tumor, and (G and H) nontumor nuclei. For the class of immunopositive tumor nuclei, there are 8 TPs, 3 FPs, 3 FNs, and 355,202 TNs. For the class of immunonegative tumor nuclei, there are 199 TPs, 35 FPs, 98 FNs, and 354,884 TNs. For the class of nontumor nuclei, there are 110 TPs, 49 FPs, 111 FNs, and 354,946 TNs. Red, green, and yellow dots represent TPs, FPs, and FNs, respectively. Magenta dots (in the right column) are gold-standard annotations that are matched with automated (B) detections and (D, F, and H) classifications.

Table 2 lists the nucleus detection and classification performance using different models. The reference baseline (untransformed) is the deep regression model trained with source data only and tested on target data. The proposed method outperforms the baseline by a large margin in terms of recall, *F*_1_ score, and AUC while providing a comparable precision. In particular, our method delivers a much higher *F*_1_ score than the baseline in classification and greatly closes the gap to the ideal supervised model trained with all real target annotations only. This suggests that models trained on one data set might not generalize to another data set, even though both use Ki-67 IHC staining. Adversarial image translation followed by deep regression modeling can improve the performance. Figure 2 shows some qualitative results of nucleus detection and classification. Confusion matrices, specificity, sensitivity, and area under the receiver operating characteristic curve are listed in Appendix Tables A1, A2, and A3. For object recognition in images, non-nucleus pixels are a dominant group, and the majority of them are correctly predicted as non-nucleus pixels. For a further comparison, we also trained a very deep regression model with the residual network (ResNet)-152^31^ as the backbone, and the results are provided in the Appendix.

**TABLE 2.**
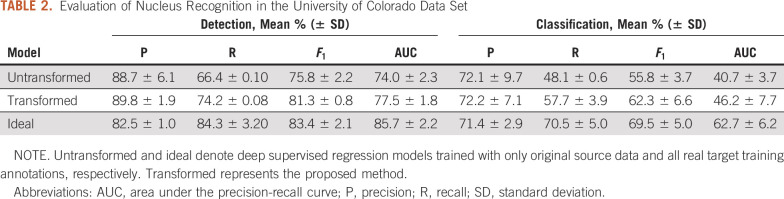
Evaluation of Nucleus Recognition in the University of Colorado Data Set

Table 3 lists the proposed method compared with multiple, popular, fully supervised deep learning models such as fully convolutional network-8s (FCN-8s),^37^ U-Net,^30^ fully convolutional regression network (FCRN) A/FCRNB,^38^ and fully residual convolutional network (FRCN),^33^ which are trained only with all real target annotations. Our method outperformed FCN-8s (by 27.7% and 18.7% in *F*_1_ score), U-Net (by 20.2% and 14.7% in *F*_1_ score), and FCRNA (by 17.9% and 6.5% in *F*_1_ score) for nucleus detection and classification, respectively, and it is competitive with FRCN, a state-of-the-art, fully supervised architecture for nucleus/cell quantification. Note that our method does not use any real target training labels for model training.

**TABLE 3.**
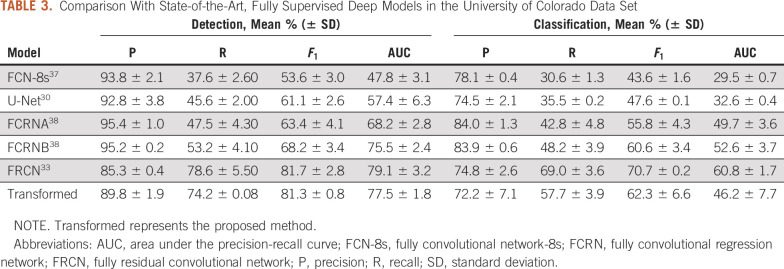
Comparison With State-of-the-Art, Fully Supervised Deep Models in the University of Colorado Data Set

**FIG 3.**
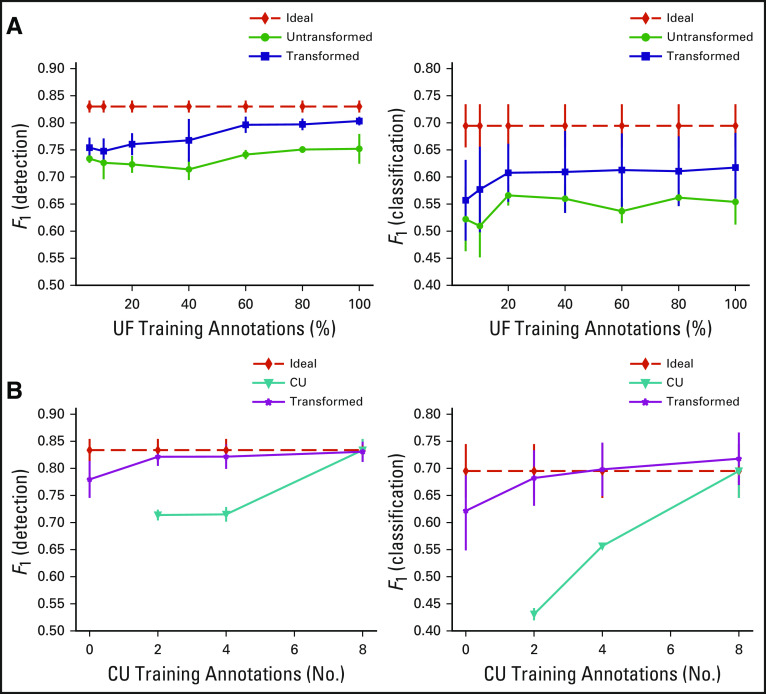
The mean and standard deviation of *F*_1_ score of nucleus detection and classification in the cross-validation with respect to different numbers of (A) source and (B) target training annotations. The *x*-axis in (B) represents the number of target training images. The red dashed lines represent the models trained with all real target annotations only. The green and cyan curves denote the models trained with different numbers of original source and target data only, respectively. CU, University of Colorado; UF, University of Florida.

Figure 3A explores the effects of the amount of annotated source training data on nucleus recognition. Translation of more source images improved the nucleus recognition performance (blue curves); however, the *F*_1_ score was inclined to saturate when using > 40% of source training data. Of note, training with converted source images always outperformed learning with original source data alone (green curves). Figure 3B shows the results from models using a mixture of 40% converted source training data and different numbers of real target training annotations (magenta curves). Similarly, using more target training data is helpful, and a small subset (eg, 4 images) may deliver equivalent performance to those using the full target training set. In addition, learning with mixed data seems to be beneficial compared with training with limited target data only (cyan curves).

**FIG A2.**
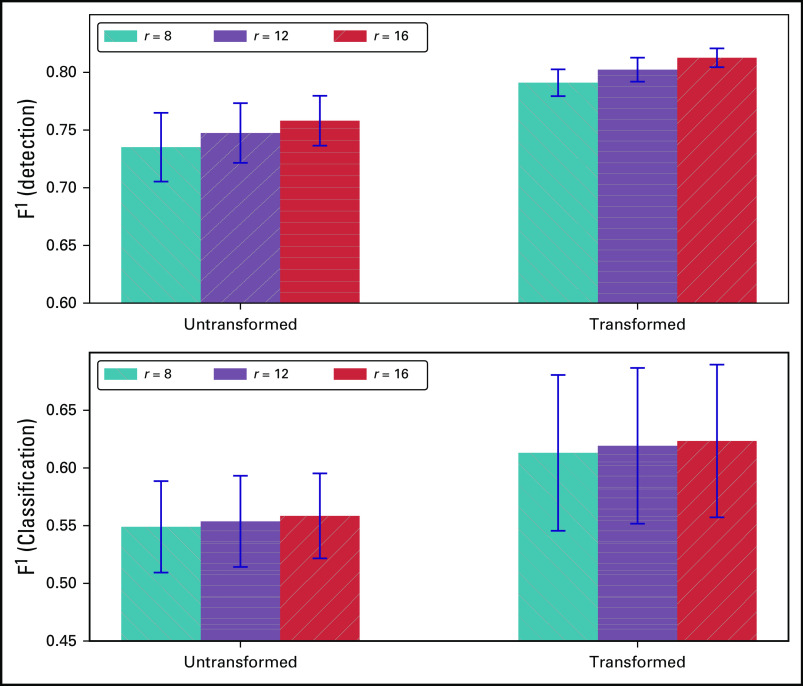
*F*_1_ score of nucleus detection and classification with different values of radius *r* used to define gold-standard areas. The blue lines denote the standard deviation of the *F*_1_ score.

After previous work,^39^ we evaluated the effects of the radius parameter *r* used to define the gold-standard areas. A smaller *r* means a more rigorous definition and higher confidence of nucleus localization. Appendix Figure A2 shows the *F*_1_ score with 3 different radii: *r* = 8, 12, and 16 pixels. We see that radius only affects performance slightly, which suggests that the proposed method produces accurate nucleus localization (ie, detected nucleus centers are close to real ones). Regardless of *r* used, our method significantly outperformed the models trained with original source data only. This confirms that domain adaptation improves performance when no target data labels are available.

## DISCUSSION

This study shows that deep learning–based domain adaptation can be applied to nucleus recognition for Ki-67 LI assessment when no target training annotations are available. Deep learning represents the state-of-the-art technology in biomedical image analysis.^11,13,14^ Many neural network architectures are proposed for image recognition^12,31,40,41^ and other image computing tasks.^10,17,18^ Most applications use these architectures as the base networks and fine-tune them toward specific tasks or target domains. However, it might be difficult to collect sufficient target training annotations for proper fine-tuning in some applications,^42^ especially in the medical imaging domain. Our method directly converts annotated source images into target-like ones and uses the converted images to train a deep regression model for nucleus recognition on real target data. This is important for Ki-67 counting because individual nucleus annotation for deep supervised model training is labor intensive. Our approach can transfer learned knowledge from one data set to another to address the issue of stain variation in Ki-67 IHC images. These experimental results show the great potential of deep learning–based domain adaptation in Ki-67 counting and can promote re-use of deep models designed for downstream supervised learning tasks.

Our study also quantifies the effects of the number of source data annotations on image translation for nucleus identification. We show that a subset of source training data (eg, 40%) can deliver competitive performance with the full data set probably because 40% of the data are sufficient to cover enough diversity of the nucleus appearance. This experiment is helpful because some data sets might be easy to collect and annotate, and a sensitivity analysis would potentially provide a guideline for data preparation. We also explored how the amount of target training data affect the performance because large-scale target data annotations are more difficult to obtain than a small subset. We find that learning with a mixture of converted source images and limited real target training annotations can compete with training on the full target data set only, which suggests that image translation is also beneficial when only limited target data are available.

In addition to the adversarial domain adaptation framework, we also present an efficient deep pixel-to-pixel network for nucleus identification, which is more streamlined than typical computerized Ki-67 scoring methods that use a multistage image processing pipeline.^43,44^ Our previous study suggested that nucleus recognition can be achieved by using an end-to-end deep neural network.^22^ Here, we tailored the previous network architecture^22^ to fit a single task, which did not require additional ROI annotations for model training. We also truncated the network into a compact and concise model such that the training process was sped up and exhibited lower memory usage. The modified network is naturally suitable for regression modeling, which has shown better performance than pixel-wise classification in nucleus localization.^38,39^ Compared with other automated methods as well as eyeball estimation and manual counting, our pixel-to-pixel model is more efficient and reproducible. Our method also provides better nucleus recognition than a previous very deep network, ResNet-based FCN,^30,31^ for most metrics.

Although WSI is widely used in digital pathology, it is far more common for pathologists to manually count Ki-67 LI in small, selected regions. However, quantitative analysis of WSI images can provide a detailed characterization of the entire tumor morphologic landscape.^45^ WSI produces gigapixel-scale images, and these images are commonly divided into a large number of small tiles that can be easily loaded for graphics processing unit computation.^10^ In the experiments, we evaluated our method on only pancreatic NET image data sets from only 2 different institutions, but this work will be expanded to include more interinstitutional data sets in the future. We do not provide uncertainty estimation of nucleus recognition in the experiments. Another potential limitation of this study might be that the gold standard was provided by a single pathologist.

In the experiments, we empirically set the hyperpara-meter values (eg, learning rate, batch size) for model training on the basis of a balance of model complexity, performance, and time cost. Meanwhile, we conducted only twofold cross-validation because of expensive com-putation for model training. However, we followed state-of-the-art methods^22,25^ to select and design network architectures. We believe that our model is effective in nucleus quantification and comparable to state-of-the-art, fully supervised models, but we are also aware that our model can be improved with the advancement of deep learning.^11,46^

In conclusion, we have developed an automated deep learning–based domain adaptation framework to quantify different types of nuclei for Ki-67 LI assessment in pancreatic NETs. It is able to provide competitive performance with state-of-the-art, fully supervised learning models and thus demonstrates the great potential of deep domain adaptation in Ki-67 counting, which can significantly reduce human effort for data annotation. Future work will focus on optimizing network architectures and applying the method to WSI analysis and more interinstitutional data.

## Figures and Tables

**TABLE A1.**
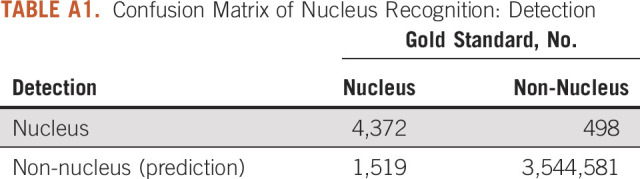
Confusion Matrix of Nucleus Recognition: Detection

**TABLE A2.**
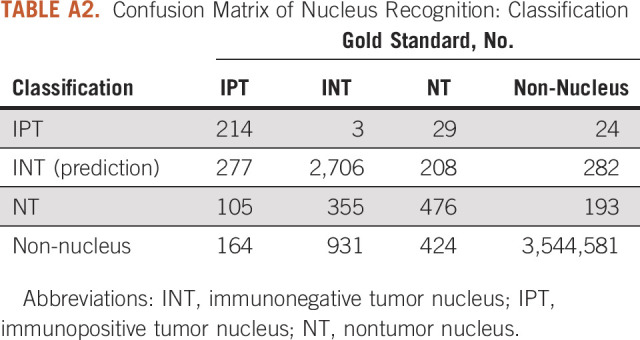
Confusion Matrix of Nucleus Recognition: Classification

**TABLE A3.**
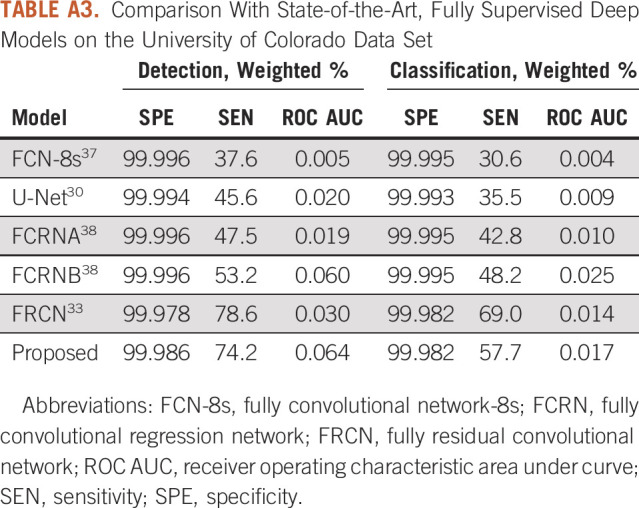
Comparison With State-of-the-Art, Fully Supervised Deep Models on the University of Colorado Data Set

## Appendix

### Mathematical Modeling for Cycle-Consistent Generative Adversarial Network

Mathematically, the cycle-consistent generative adversarial network (GAN) can be formulated as follows (Eqs A1-A4)^25^:

$$arg\underset{G_{st},G_{ts}}{\min}\underset{D_{s},D_{t}}{\max}{ℒ}_{GAN}\left(G_{st},D_{t}\right)+{ℒ}_{GAN}\left(G_{ts},D_{s}\right)+\lambda {ℒ}_{cycle}\left(G_{st},G_{ts}\right)$$ (A1)

$${ℒ}_{GAN}\left(G_{st},D_{t}\right)=E_{x^{t}∼X^{t}}\left[\text{log }D_{t}\left(x^{t}\right)\right]\;+\;E_{x^{s}∼X^{s}}\left[\left(1-\text{log }D_{t}\left(G_{st}\left(x^{s}\right)\right)\right)\right]$$ (A2)

$${ℒ}_{GAN}\left(G_{ts},D_{s}\right)=E_{x^{s}∼X^{s}}\left[log\;D_{s}\left(x^{s}\right)\right]\;+\;E_{x^{t}∼X^{t}}\left[\left(1-log\;D_{s}\left(G_{ts}\left(x^{t}\right)\right)\right)\right]$$ (A3)

$${ℒ}_{cycle}\left(G_{st},G_{ts}\right)=E_{x^{s}∼X^{s}}\left[||G_{ts}\left(G_{st}\left(x^{s}\right)\right)-x^{s}|{|}_{1}\right]\;+\;E_{x^{t}∼X^{t}}\left[||G_{st}\left(G_{ts}\left(x^{t}\right)\right)-x^{t}|{|}_{1}\right]$$ (A4)

where *λ* ≥ 0 is a hyperparameter to weight the cycle consistency, E represents the expectation, and ‖·‖_1_ denotes the I_1_ norm. During the optimization of Equation A1, the generators and discriminators are alternatively updated until a balance is achieved.

### Mathematical Modeling for Deep Regression

Mathematically, the deep regression model *R* can be formulated as follows (Eq A5):

$${ℒ}_{reg}\left(G_{st},R\right)=E_{\left(x^{s},y^{s}\right)∼\left(X^{s},Y^{s}\right)}\left[||{\left(y^{s}+\alpha {\bar{y}}^{s}1\right)}^{\frac{1}{2}}⊙\left(R\left(G_{st}\left(x^{s}\right)\right)-y^{s}\right)|{|}_{F}^{2}+||{\left(y^{s}+\alpha {\bar{y}}^{s}1\right)}^{\frac{1}{2}}⊙\left(R\left(x^{s}\right)-y^{s}\right)|{|}_{F}^{2}\right],$$ (A5)

where the label *y^s^* is a 3-channel proximity map that measures the proximity of pixels to their closest same-class nucleus centers, with 1 channel for each nucleus subtype.^22^ ${\bar{y}}^{s}$ represents the channel-wise mean of *y^s^*, and 1 is a 3-dimensional matrix with all elements being 1. *α* = 5 is a contribution controller for different image regions, ‖·‖_F_ denotes the Frobenius norm applied to each channel, and ⊙ indicates the element-wise multiplication.

### Training Details

For the University of Colorado (CU) data set, we had 28 training images with 8 annotated, 8 validation images with 2 annotated, and 36 test images with 10 labeled. The CU images (40× magnification) were resized by a factor of 0.5 to match the 20× magnification of the University of Florida (UF) images for deep regression model training and testing. Within each iteration of regression model training, we randomly cropped and fed 220 × 200 × 3 patches into the proposed U-Net–like network.

We set *λ* = 10 in Equation A1 and *η* = 0.5 for pixel suppression during testing. We used the Adam algorithm (Kingma DB, et al: Proc Int Conf Learn Representations, 2015) to train the cycle-consistent GAN, with learning rate = 2 × 10^−4^ and number of epochs = 170. We trained the deep regression model with stochastic gradient descent with Nesterov momentum (Sutskever I, et al: Proc Int Conf Mach Learn, 2013) and set the parameter values as momentum 0.99, learning rate = 10^−3^, batch size = 4, weight decay = 10^−6^, and number of iterations = 10^5^. We scaled the proximity map by a factor of 5 to facilitate training^33^ and stopped the training if the performance on the validation set did not improve for 2 × 10^4^ iterations. We implemented the proposed method with PyTorch (PyTorch: https://pytorch.org) on a workstation with a GeForce GTX 1080 Ti graphics processing unit (Nvidia, Santa Clara, CA).

### Comparison With Deep Residual Networks

Following the work of fully convolutional networks (FCNs),^37^ we used residual network (ResNet)-152 as the backbone to generate a pixel-to-pixel FCN by removing the global average pooling layer and the final fully connected layer and then adding an upsampling layer (implemented as bilinear interpolation) to produce dense prediction. We trained this very deep ResNet-based FCN with the proposed regression loss (ie, Eq A5). The performance of this network on the test set is 93.8% precision, 51% recall, 66% *F*_1_ score, and 69.5% area under the precision-recall curve (AUC) for detection and 79.2% precision, 44.4% recall, 55.8% *F*_1_ score, and 49.7% AUC for classification. Most of these metric values are lower than that of our U-Net–like architecture. This might be a result of our network having long-range skip connections, which can take advantage of high-resolution information in low layers for precise nucleus localization, and multilevel context aggregation connections, which can handle scale variation of nuclei.

### Data Collection

The 2 data sets were collected from 2 separate institutions, UF and CU Anschutz Medical Campus. UF is a public, land-grant, sea-grant, and space-grant research university. It is home to 16 academic colleges and > 150 research centers and institutes. Currently, UF has > 55,000 students enrolled. The CU Anschutz Medical Campus is the largest academic health center in the Rocky Mountain region and a world-class medical destination at the forefront of transformative education, science, medicine, and health care. The campus includes the CU health professional schools; multiple centers and institutes; and 2 nationally ranked hospitals, CU Hospital and Children’s Hospital Colorado, which treat nearly 2 million patients each year. The campus currently has 4,500 students enrolled.

For the CU data set, cases from CU were identified by searching the anatomic pathology laboratory information system for the date range January 1, 2006, to January 30, 2018, which met the following criteria: Ki-67 immunostain was performed, part type of pancreas resection or Whipple resection, and diagnostic text that included “neuroendocrine.” Slides were requested from the CU Biorepository Core Facility. Of note, some cases were incomplete or not available from the archive. Ki-67 slides from the retrieved cases were digitized on an Aperio ScanScope AT2 slide scanner (Leica Biosystems, Vista, CA) at ×40 equivalent magnification (0.252 μm/pixel) and eventually scaled by 50%. Only cases with 3′-diaminobenzidine (DAB; brown) detection of Ki-67 were included in the study (eg, 3-amino-9-ethylcarbazole/AEC-red detection was excluded). Ki-67 whole-slide images were reviewed in ImageScope (Leica Biosystems) by an expert GI pathologist, and a single region of interest (ROI) measuring 300 × 300 μm was drawn to include the hot spot (ie, the area of tumor estimated to have the highest Ki-67 labeling index). Custom software written in Python 2 and using the Openslide library (Goode A, et al: J Patho Inform 4:27, 2013) was then used to read the annotation file and extract the ROIs from the wholes-slide image base layer as uncompressed tagged image file format files. Twenty of the ROIs were then chosen to provide a range of Ki-67 labeling indices.

### Gold-Standard Generation

We developed a computer-aided annotation tool for individual nucleus labeling using the MATLAB (MathWorks, Natick, MA) programming language. The tool provides a user interface such that the user can use the mouse to label nuclei by clicking a point at the center of each nucleus. Different types of nuclei are labeled with different colors. T.C.C., who is a board-certified and practicing GI pathologist, selected the regions of hot spots and conducted the nucleus annotation in Ki-67–stained images.

### Explanation of Terminology/Algorithms

#### Domain adaptation.

Given a source domain and a target domain, domain adaptation aims to improve the learning of the target predictive function using the knowledge from the source domain.

#### Generative adversarial network.

A GAN^24^ is a neural network architecture that typically consists of a generator and a discriminator. The generator learns to create images to fool the discriminator, while the discriminator is optimized to distinguish between real and generated images.

#### Cycle-consistent GAN.

Cycle-consistent GAN^25^ is a neural network architecture that consists of 2 generator-discriminator pairs, each corresponding to 1 domain or data set. It introduces a cycle-consistent loss into the standard GAN framework such that the reconstructions of converted images are identical to their original versions.

#### PatchGAN.

PatchGAN^29^ is a traditional convolutional neural network used as a discriminator in adversarial learning, which aims to classify whether image patches are real or fake. The network is run convolutionally across the entire image and can be applied to arbitrary-sized images. Compared with a full-image discriminator, PatchGAN has fewer parameters and runs faster.

#### Residual learning block.

Residual learning block^31^ is a building unit used to construct deep neural networks. The block consists of a small feedforward neural network, which fits a residual mapping, and a shortcut connection, which realizes an identity mapping. These 2 mappings are summed to recast the original, underlying mapping. In our architecture, the decoder consists of 4 stacked residual blocks, and each block contains 2 sets (the first block has only 1) of convolution-bn-elu operations, where bn and elu denote batch normalization and exponential linear unit, respectively. A stride-2 convolution is used to connect 2 residual blocks for feature map downsampling. The decoder is also composed of 4 residual blocks, but a transposed convolution is exploited to upsample feature maps.
